# Beam steering at the nanosecond time scale with an atomically thin reflector

**DOI:** 10.1038/s41467-022-29976-0

**Published:** 2022-06-14

**Authors:** Trond I. Andersen, Ryan J. Gelly, Giovanni Scuri, Bo L. Dwyer, Dominik S. Wild, Rivka Bekenstein, Andrey Sushko, Jiho Sung, You Zhou, Alexander A. Zibrov, Xiaoling Liu, Andrew Y. Joe, Kenji Watanabe, Takashi Taniguchi, Susanne F. Yelin, Philip Kim, Hongkun Park, Mikhail D. Lukin

**Affiliations:** 1 Department of Physics, Harvard University, Cambridge, MA 02138 USA; 2 Max Planck Institute of Quantum Optics, Hans-Kopfermann-Straße 1, D-85748 Garching, Germany; 3 ITAMP, Harvard-Smithsonian Center for Astrophysics, Cambridge, MA 02138 USA; 4 Department of Chemistry and Chemical Biology, Harvard University, Cambridge, MA 02138 USA; 5 Department of Materials Science and Engineering, University of Maryland, College Park, MD 20742 USA; 6 Research Center for Functional Materials, National Institute for Materials Science, 1-1 Namiki, Tsukuba, 305-0044 Japan; 7 International Center for Materials Nanoarchitectonics, National Institute for Materials Science, 1-1 Namiki, Tsukuba, 305-0044 Japan; 8 John A. Paulson School of Engineering and Applied Sciences, Harvard University, Cambridge, MA 02138 USA

**Keywords:** Two-dimensional materials, Nanoscale devices, Photonic devices

## Abstract

Techniques to mold the flow of light on subwavelength scales enable fundamentally new optical systems and device applications. The realization of programmable, active optical systems with fast, tunable components is among the outstanding challenges in the field. Here, we experimentally demonstrate a few-pixel beam steering device based on electrostatic gate control of excitons in an atomically thin semiconductor with strong light-matter interactions. By combining the high reflectivity of a MoSe_2_ monolayer with a graphene split-gate geometry, we shape the wavefront phase profile to achieve continuously tunable beam deflection with a range of 10°, two-dimensional beam steering, and switching times down to 1.6 nanoseconds. Our approach opens the door for a new class of atomically thin optical systems, such as rapidly switchable beam arrays and quantum metasurfaces operating at their fundamental thickness limit.

## Abstract (web summary)

Andersen et al. have demonstrated a new type of beam steering device based on the excitonic response of an atomically thin semiconductor. Using electrostatic gates, the authors achieved tunable steering with switching times on the nanosecond scale.

## Introduction

Conventional optical devices, typically made from materials with relatively weak light-matter interactions and a smooth optical response on the wavelength scale, are bulky to accumulate the desired effect on the optical wavefront. Recent advances in flat optics demonstrate that steep gradients in the phase, amplitude, or polarization can be used to control light fields on sub-wavelength scales^1–3^, enabling novel optical phenomena and applications, including ultrathin lenses^4,5^, metasurfaces^6^, non-reciprocity^7^, and negative refraction^8^.

While most demonstrations of flat optical elements so far have involved passive devices, their active counterparts have recently attracted great interest^9–12^. Tuning mechanisms including optically^13^ and thermally^14^ induced phase transitions, as well as magnetically tuned transparency in magneto-plasmonic crystals^15^, have been demonstrated. Micro-electrical mechanical systems (MEMS) technology has also been employed to spatially modulate the optical response^16^, but the operation speed of such devices is typically limited to the kHz or few MHz range. A promising avenue to overcome this limitation involves full electrical control, via for instance ionic^17^ or electrostatic^18–20^ gating. In order to achieve fully programmable devices, a key challenge is to achieve fast, continuous tunability of multiple independent channels.

**Fig. 1:**
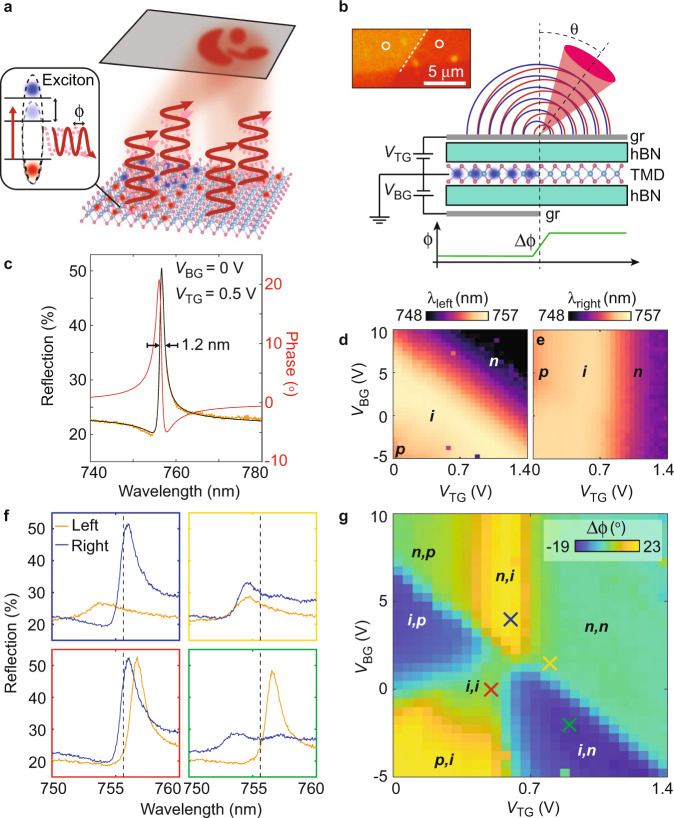
Continuously tunable phase patterning in a van der Waals heterostructure. **a** Schematic of our approach: patterned electrostatic doping of atomically thin transition metal dichalcogenides (TMDs) allows for spatial control of the exciton resonance (inset). Thus, a continuously tunable phase profile is imparted on the reflected wavefront, enabling wide-ranging possibilities for beam control. **b** Schematic of SG-FET structure. Since the bottom gate only covers part of the device, the phase can be tuned independently in the two sides. The phase discontinuity in the reflected wavefront causes the two halves to constructively interfere at an angle in the far field. Inset: zoomed-in optical microscope image of the device, with gate edge indicated by white dashed line. **c** Representative reflection spectrum (orange) from left side of gate edge in intrinsic regime (*V*_BG_ = 0 V and *V*_TG_ = 0.5 V), with asymmetric resonance fit (black), which allows for extracting the phase (red). **d**, **e** Gate dependence of *λ*_left_ and *λ*_right_, respectively (locations indicated by circles in inset of **b**). The exciton resonance blue-shifts upon electrostatic doping. While *λ*_left_ depends on 8*V*_TG_+ *V*_BG_, *λ*_right_ is largely independent of V_BG_. The intrinsic regime appears at an offset of *V*_TG_ = 0.5 V, likely due to charge collection at the top gate. The small voltage range of the intrinsic regime suggests some doping via in-gap states. **f** Reflection spectra from left (orange) and right (blue) side of gate edge at different gate voltage combinations shown as correspondingly colored crosses in **g**. Dashed gray line indicates *λ*_0_ = 755.6 nm. **g** Gate dependence of phase difference Δ*ϕ* between the right and left side at *λ*_0_ = 755.6 nm, computed from fits as in (**c**). A tunable Δ*ϕ*-range of 42° is achieved. Large positive Δ*ϕ* is achieved when *λ*_left_ < *λ*_0_ < *λ*_right_ (blue cross), while large negative Δ*ϕ* is achieved when *λ*_right_ < *λ*_0_ < *λ*_left_ (green). Δ*ϕ* is closer to zero when either both sides are doped (*λ*_left_, *λ*_right_ < *λ*_0_; yellow) or both are intrinsic (*λ*_0_ < λ_left_, *λ*_right_; red).

To address this challenge, we realize a continuously tunable, atomically thin optical device based on phase profile modulation in field-effect transistors composed entirely of two-dimensional van der Waals materials. The optically active element of our system is exfoliated monolayer MoSe_2_—an atomically thin semiconductor that hosts tightly bound excitons in the optical (visible) domain. Excitons in TMDs have been widely proposed as an appealing system for quantum optical devices^21^, due to their quantum coherence properties^22^ and potential for quantum nonlinear effects mediated by confinement-enhanced exciton–exciton interactions^23^. In high-quality exfoliated flakes encapsulated in boron nitride (hBN), these excitons can exhibit very strong light-matter interaction, enabling almost perfect reflection from an atomically thin reflector^24–26^. By employing near-transparent graphene gates, the exciton resonance in TMDs can be electrostatically tuned^27,28^. Due to the two-dimensional nature of TMDs, the exciton resonance is tuned throughout the whole material, circumventing the effects of screening commonly encountered in bulk semiconductors^29^. These features allow for spatially dependent control of the phase and amplitude of the reflected light, which modifies the beam in the far-field due to interference, enabling wide-ranging possibilities for optical beam control (Fig. 1a).

## Results

### Phase modulation through electrostatic gating of excitons

We demonstrate this approach by realizing fast, continuously tunable beam steering with a split-gate geometry (Fig. 1b). Using the dry-transfer technique, we assemble our device (Fig. 1b, inset) from exfoliated flakes of monolayer MoSe_2_, graphene (top and bottom gates), and hBN (gate dielectric) into a split-gate field-effect transistor (SG-FET) structure, in which the bottom gate (BG) only covers part of the device. The gate geometry enables independent electrostatic doping of the two parts of the device, and—because the exciton resonance shifts with doping—a very steep phase gradient near the gate edge. The non-zero width of this step is due to stray electric fields and is comparable to the thickness of the gate dielectric (~50 nm). By focusing light on the gate-edge, the two halves of the reflected wavefront gain a different phase (Δ*ϕ*) and thus constructively interfere in the far-field at an angle to the optical axis (Fig. 1b).

Figure 1c shows the absolute reflectance spectrum, collected away from the gate edge in the intrinsic regime (*T* ~ 6 K). The MoSe_2_ features a sharp excitonic resonance at *λ*_intrinsic_ ~ 757 nm, with a linewidth of 1.2 nm. The asymmetric line-shape is attributed to the interference between the Lorentzian exciton reflection and light reflected from other interfaces in the device^26^ (Supplementary Notes I and II). By fitting the asymmetric resonance profile (black; Supplementary Note I), we extract the phase of the reflected light (red). The phase of the exciton reflection itself changes by 180° (*π*) across the resonance, but the interference with the background reflections reduces the overall phase range (we note that this is not an inherent limitation; see Supplementary Note II and Supplementary Figs. S1–S3).

In order to tune the phase at a given wavelength, we shift the exciton resonance by electrostatically doping the MoSe_2_ with the top and bottom gates (*V*_TG_ and *V*_BG_, respectively). Figure 1d, e shows the gate dependence of the resonance wavelengths, *λ*_left_ and *λ*_right_, on the left and right sides of the gate edge, respectively. The exciton resonance blue-shifts by several linewidths in the *p*- and *n*-doped regimes, resulting from bandgap renormalization and repulsive polaron formation^26,28,30^. In the left (dual-gated) side of the device (Fig. 1d), the resonance depends on both *V*_TG_ and *V*_BG_; more specifically the weighted sum, 8*V*_TG_ + *V*_BG_, due to unequal top and bottom hBN thicknesses. In contrast, the exciton resonance on the right (single-gated) side of the device only depends on *V*_TG_, except for a slight *V*_BG_-dependence of the onset of the *p*-doped regime due to contact activation (Fig. 1e). These distinct dependencies of *λ*_left_ and *λ*_right_ on the gate voltages allow for tuning their relative positioning (Fig. 1f) and are key to creating the abrupt phase discontinuity.

Fitting the spectra at all gate voltage combinations in Fig. 1d, e, we find the gate dependence of the phase difference (Δ*ϕ*) between the two sides of the device at *λ*_0_ = 755.6 nm (Fig. 1g), indicating a continuously tunable range of 42°. The wavelength is chosen to be blue-detuned relative to the intrinsic exciton resonance, such that the exciton resonance is swept through *λ*_0_ upon electrostatic gating. Inspecting Fig. 1g, we identify four regimes that are central to the operation of our system, distinguished by the relative positioning of *λ*_left_ and *λ*_right_: a large positive phase difference is achieved when the resonance on the left side is blue-shifted past *λ*_0_, while *λ*_right_ is kept red-shifted (Fig. 1f, blue box). Conversely, a large negative phase difference is realized when *λ*_right_ < *λ*_0_ < *λ*_left_ (Fig. 1f, green box). Finally, the magnitude of the phase difference is much smaller when the two sides are either both doped (Fig. 1f, yellow box) or both intrinsic (Fig. 1f, red box). As can be seen in Fig. 1f, the doping induced blue-shift is accompanied by a decrease in amplitude, reducing the maximum phase of the combined reflection in the doped regimes.

### One-dimensional beam steering

**Fig. 2:**
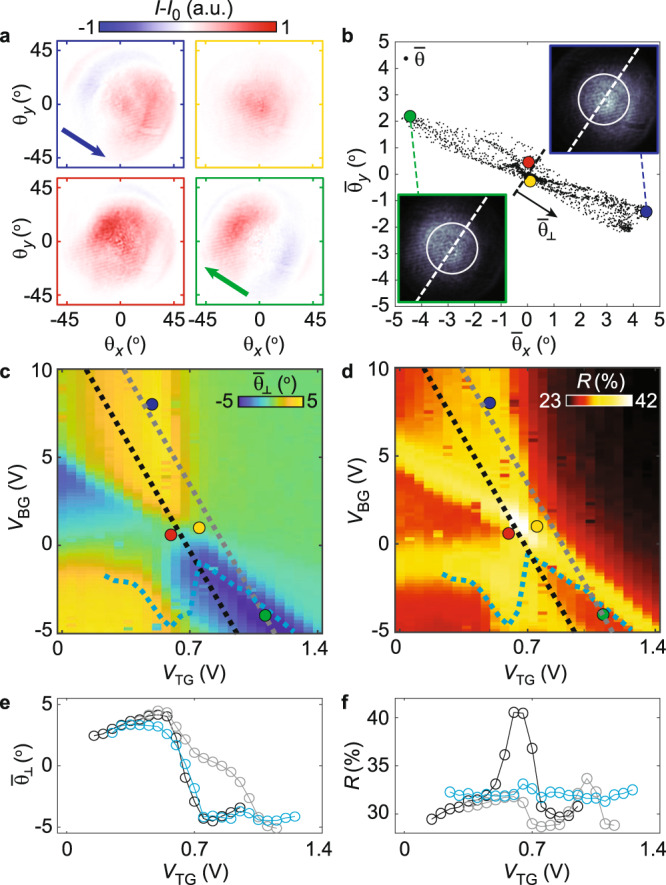
Continuously tunable beam steering. **a** Fourier images of reflected beam (*λ*_0_ = 755.6 nm) in the four regimes after subtracting the reflection in the highly doped regime (*V*_BG_ = 10 *V* and *V*_TG_ = 1.4 V). When the exciton resonance is blue-shifted past *λ*_0_ in only one side of the device (blue, green), the beam is deflected away from that side. If neither or both are blue-shifted past *λ*_0_, the phase difference is small and little deflection is observed (red, yellow). **b** Scatter plot of the beam deflection (${\bar{\theta }}_{x},{\bar{\theta }}_{y}$) for the full range of gate voltages, showing that the deflection is perpendicular to the gate edge (dashed line) and continuously tunable. Inset: Fourier images without background subtraction. **c** The gate dependence of the deflection perpendicular to the gate edge (${\bar{\theta }}_{\perp }$) is in very good agreement with that of the phase difference shown in Fig. 1g. **d** Gate dependence of reflection amplitude. Regions with high reflection indicate that one of the resonances crosses through *λ*_0_. **e**, **f** Linecuts indicated by dashed black, teal, and gray lines in (**c**) and (**d**), respectively, highlighting the continuous steering capability and that the reflection can be kept relatively constant while deflecting the beam. Connecting lines in **e** and **f** are guides to the eye.

Having demonstrated independent phase tunability in the two sides of the device, we next measure the beam steering capabilities by focusing the laser beam (*λ*_0_ = 755.6 nm; numerical aperture, NA = 0.75) onto the gate edge (see Supplementary Note III for alignment procedure) and imaging the reflected beam in the Fourier plane (see Supplementary Video S1). The Fourier plane polar coordinates *r*_F_ and *ϕ*_F_ are converted to angular deflection via *θ* = sin^−1^ (r_F_/*f*), where *f* is the focal length of the objective, and decomposed into *θ*_*x*_ = *θ* · cos(*ϕ*_F_) and *θ*_*y*_ *=* *θ* · sin(*ϕ*_F_). The undeflected beam is approximately Gaussian with an angular width (standard deviation) of 17°. Figure 2a shows representative Fourier images in the four regimes identified in Fig. 1f, g, after subtracting the reflected intensity without exciton effects (*I*_0_), obtained by heavily doping the device (*V*_BG_ = 10 V, *V*_TG_ = 1.4 V). Defining the center-of-mass deflection of the full (not background-subtracted) reflection as ${\bar{\theta }}_{i}=\frac{\Sigma I{\theta }_{i}}{\Sigma I}$, we present scatter plots of ${\bar{\theta }}_{x}$ and ${\bar{\theta }}_{y}$ for the full range of gate voltages in Fig. 2b, including highlighted deflections in the four regimes (colored circles) and Fourier images without background subtraction (insets).

We find that the reflection is deflected in the expected direction, perpendicular to the gate edge. The tunable deflection range is 9.8°, in very good agreement with theoretical predictions based on the phase difference range observed in Fig. 1g. Specifically, for a diffraction-limited beam spot, the deflection is predicted to be ${\bar{\theta }}_{\perp }=\frac{{{{{{\rm{NA}}}}}}}{0.42\cdot \sqrt{2{\pi }^{3}}}\cdot \Delta \phi =0.23\cdot \Delta \phi$, which gives a range of 9.7° (Supplementary Note IV). Moreover, the gate dependence of the deflection (Fig. 2c) closely resembles that of the extracted phase difference (Fig. 1g), consistent with the deflection arising due to the sharp phase discontinuity imparted on the wavefront. We identify distinct steering behavior in the four regimes: when only one side of the device is kept red-shifted relative to *λ*_0_ (blue and green boxes in Fig. 2a), the beam is deflected towards that side. Intuitively, the light from the two sides interferes constructively when the path length is shorter for the side with the greater phase. In the two other regimes, on the other hand, where the two sides are either both blue-shifted past *λ*_0_ (yellow) or both red-shifted (red), we observe near-zero deflection, consistent with a much smaller phase difference. We note that very similar behavior, although with a smaller deflection range, was observed for *λ*_0_ down to 752 nm.

Figure 2d shows the gate dependence of the integrated reflection. The different regimes are separated by regions of strong reflection, since these indicate that one of the resonances crosses through *λ*_0_. While the four different regimes are easily identified, we emphasize that the phase difference, and thus the beam deflection, is tuned continuously, as shown in Fig. 2b. The continuous tunability is highlighted in Fig. 2e, showing linecuts from Fig. 2c. Although the reflection amplitude is gate dependent (Fig. 2d, f), the reflection variations can be reduced substantially while still keeping a similar deflection range by avoiding the combined resonance (gray linecut), or even designing a near-iso-reflection path in voltage space (teal).

### Two-dimensional beam steering

**Fig. 3:**
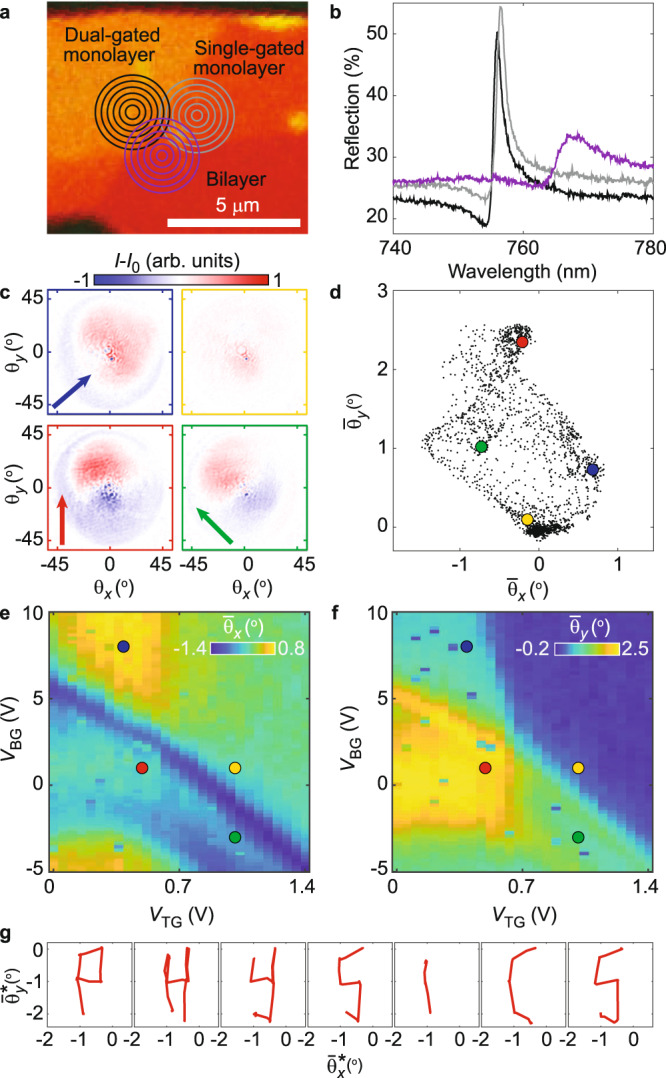
Two-dimensional beam steering. **a** Zoomed-in optical image of device, indicating regions of monolayer and bilayer MoSe_2_, as well as gate coverage. By tuning the relative phase and amplitude of the reflection from the three regions, more intricate wavefront phase profiles can be achieved. **b** Reflection spectra from the dual-gated monolayer (black), single-gated monolayer (gray) and the bilayer (purple) in the intrinsic regime (*V*_BG_ = 0 V and *V*_TG_ = 0.5 V). Since the resonance is red-shifted in the bilayer, it acts as a dielectric (non-resonant) reflector at the wavelengths used here. **c** Fourier images of reflected beam (*λ*_0_ = 754.1 nm) in the four regimes after subtracting reflection in the highly doped regime (*V*_BG_ = 10 *V* and *V*_TG_ = 1.4 V). The beam is now steered in two dimensions. Red: when both monolayer resonances are red-shifted relative to *λ*_0_, their phase is higher than in the bilayer region, causing the beam to deflect upwards. Blue and green: when one of the monolayer resonances is kept red-shifted relative to *λ*_0_, the beam is deflected towards that monolayer region. **d** Scatter plot of the center-of-mass deflection $({\bar{\theta }}_{x},{\bar{\theta }}_{y})$, with the points from (**c**) highlighted. The set of beam deflections now span a two-dimensional area. **e**, **f** Gate dependence of ${\bar{\theta }}_{x}$ (**e**) and ${\bar{\theta }}_{y}$ (**f**). While the gate dependence resembles that of the phase in Fig. 1g, ${\bar{\theta }}_{x}$ and ${\bar{\theta }}_{y}$ are now less coupled. **g** Center-of-mass deflection tracing out “PHYSICS” (rotated 148° counter-clockwise) by applying a sequence of gate voltage combinations.

In order to achieve more advanced control of the wavefront profile, we utilize the device region where the edge of the bottom gate intersects a border between monolayer and bilayer MoSe_2_ (Fig. 3a). Since the exciton resonance in bilayers is red-shifted to ~767 nm due to interlayer hybridization effects^31^ (Fig. 3b), the bilayer acts as a non-resonant dielectric reflector, thus enabling control of the relative phase between the three regions. Figure 3c shows representative Fourier images of the reflected light in the four different regimes at *λ*_0_ = 754.1 nm. When both the monolayer resonances are red-shifted relative to *λ*_0_, the beam is deflected upwards (red), as opposed to the near-zero deflection observed in Fig. 2. Keeping only one of the resonances red-shifted causes the beam to deflect upwards at an angle towards the red-shifted side (green and blue). This is further shown by plotting the full set of center-of-mass deflections, ${\bar{\theta }}_{x}$ and ${\bar{\theta }}_{y}$ (Fig. 3d); while these were clustered around a line perpendicular to the gate edge in Fig. 2b, we now observe that they span a broader, two-dimensional area. Consequently, the gate dependence of ${\bar{\theta }}_{x}$ and ${\bar{\theta }}_{y}$ (Fig. 3e, f)—while still resembling that of the phase in Fig. 1g—is now more intricate. Instead of simply being (negatively) proportional to each other, ${\bar{\theta }}_{x}$ takes on both positive and negative values when ${\bar{\theta }}_{y}$ is intermediate, and ${\bar{\theta }}_{y}$ can be either large or near-zero when ${\bar{\theta }}_{x}$ is near zero. To demonstrate the two-dimensional steering capability, we write a desired two-dimensional pattern with the center-of-mass of the reflected beam (Fig. 3g), by successively applying gate voltage combinations corresponding to the appropriate beam deflections.

The two-dimensional deflection behavior is well understood by considering the third reflection source from the bilayer region. When both the monolayer resonances are kept red-detuned relative to *λ*_0_, the phase is higher than in the bilayer region, thus imparting an upwards phase gradient on the reflected wavefront. Similarly, if only one of the monolayer regions is kept red-shifted, the phase gradient points towards that region. Hence, the three reflection sources enable two-dimensional beam control.

### Switching on the nanosecond scale

**Fig. 4:**
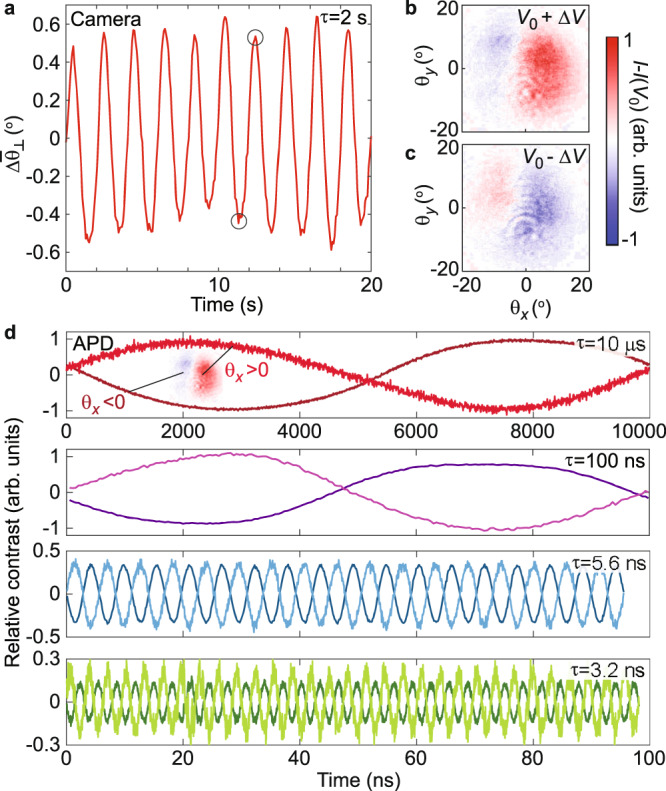
High-frequency beam steering. **a** Oscillations of center-of-mass deflection (*λ*_0_ = 754.5 nm) induced by oscillating back gate voltage (offset, *V*_0_ = 0.7 V; amplitude, Δ*V* = 0.45 V; period, *τ* =2 s) and *V*_TG_ = 0.64 V. **b**, **c** Fourier plane images collected at *V*_BG_ =*V*_0_ + Δ*V* (**b**) and *V*_BG_ = *V*_0_ − Δ*V* (**c**), as indicated by circles in **a**, after subtracting reflection at *V*_BG_ = *V*_0_. An inverted telescope was used to shrink the beam to simplify the subsequent APD measurements. **d** Photon count oscillations measured with APD at *τ* = 10 μs, 100 ns, 5.6 ns, and 3.2 ns (top to bottom). Darker (lighter) shade curves show photon counts from left (right) side of Fourier plane, as indicated by the inset in the top panel. All curves are normalized to the corresponding contrast at *τ* = 10 μs.

We investigate the temporal response of our system by applying a small oscillating bottom gate voltage, ${V}_{{{{{{\rm{BG}}}}}}}\left(t\right)={V}_{0}+\Delta V\cdot {{\sin }}\left(\frac{2\pi t}{\tau }\right)=0.7{{{{{\rm{V}}}}}}+0.45{{{{{\rm{V}}}}}}\cdot {{\sin }}\left(\frac{2\pi t}{\tau }\right)$ and a constant top gate voltage *V*_TG_ = 0.64 V, where *τ* is the period (corresponding to twice the switching time). Focusing the beam at the gate edge, as in Fig. 2, we first measure the beam deflection using a long period (*τ* = 2 s) compatible with our camera (Fig. 4a). Figure 4b, c shows the change in reflection from *V*_BG_ = *V*_0_ to *V*_BG_ = *V*_0_ + Δ*V* and *V*_BG_ = *V*_0_ − Δ*V*, respectively. Next, we measure the optical response at much higher frequencies by collecting the reflected light in the Fourier plane with an avalanche photon detector (APD; Fig. 4d). In order to ensure that we probe beam steering, as opposed to simply changes in reflection amplitude, we separately collect photons from the left (*θ*_*x*_ < 0) and right parts (*θ*_*x*_ > 0) of the Fourier plane (inset in top panel of Fig. 4d). These signals are found to oscillate with a near-180° phase difference, unambiguously indicating high-frequency beam deflection. Notably, we observe clear oscillations all the way down to a period of *τ* = 3.2 ns (*ω* = 2*π* ⋅ 0.32 GHz). Normalizing the oscillations in APD counts to those at *τ* = 10 μs, we find that the amplitude is approximately unaffected at *τ* = 100 ns, and reduced by ~60% (~80%) at *τ* = 5.6 ns (3.2 ns). Measurements of the high-frequency transmission of electrical connectors leading up to the device indicate that more than half of this reduction is in fact not due to the device itself, but rather external losses and reflections (Supplementary Note V and Supplementary Fig. S7). Reduction of pixel size and further improvement of contact quality can likely enable switching times down to a few tens of picoseconds^32,33^, ultimately limited by the MoSe_2_ mobility.

## Discussion

While the above results were obtained at a device temperature of *T* = 6 K, almost identical data were obtained at liquid nitrogen temperatures (80 K; Supplementary Note VI and Supplementary Fig. S8). Moreover, deflection was still clearly observed, although with a smaller amplitude, at a temperature easily attainable with a Peltier cooler (*T* = 230 K), and some deflection effects were even observed at room temperature. The deflection range could be further increased at all temperatures through improvements in material quality or further optimization of the hBN thicknesses and substrate permittivity. In fact, while background reflections often reduce the phase range, optimization of their phase and amplitude can increase the phase range to 2π (Supplementary Note II and Supplementary Figs. S1–S3). Combined with the introduction of more pixels in next-generation devices, this is expected to enable a larger ratio between the steering and beam divergence angles.

These observations demonstrate that our system can be an attractive platform for applications involving high-speed active optics, with on-chip integrability and potential for flexible transparent optics as very appealing features. In addition to the two-dimensional beam steering demonstrated here, our system can be scaled up into more advanced pixel arrays with feature sizes well below 100 nm through standard etch techniques, to enable a broad variety of other atomically thin optical elements, including atomically flat holograms with many controllable outputs and flat lenses with tunable focal length. In particular, our approach of using gates in two planes makes it possible to generate a 2D grid of *n* × *n* pixels (2*n* independent input channels) by etching the top and bottom gates into thin perpendicular strips (see Supplementary Note VII and Supplementary Fig. S9 for further details). The use of nanoelectrode arrays is another promising upscaling route. While exfoliated flakes with areas typically exceeding 100 μm^2^ can fit a high number of pixels, recent progress in chemical vapor deposition (CVD) growth^34^ can enable scaling up to even larger devices.

Importantly, our approach also offers unique features which could unlock quantum applications that are inherently impossible with conventional spatial light modulator (SLM) technologies. In particular, quantum analogs of metasurfaces that could generate and manipulate entangled states of light have recently been proposed^35,36^. The realization of such devices relies on creating metasurfaces from locally tunable materials that can exist in a superposition of states with different reflectivity and that facilitate quantum nonlinear effects, thus calling for the exploration of new material systems for flat optics. In contrast to materials commonly used in conventional SLMs, the K/K’ valley exciton species employed here has been widely shown to exhibit promising, gate-tunable quantum coherence properties and can be readily excited to a quantum superposition of states that interact only with left- and right-handed circularly polarized light. Moreover, the use of excitons confined in an atomically thin material renders it a promising approach for achieving quantum nonlinear effects mediated by exciton–exciton interactions. Recent works suggest that strong exciton–exciton interactions could be achieved in our system through reflective substrate engineering^23^ or by using Rydberg exciton states^37^, making active flat optics based on TMDs a very appealing platform for the investigation of quantum optical metasurfaces^35^.

## Methods

### Device fabrication

In order to minimize contact resistance, crucial to high-frequency operation, we fabricated bottom contacts to the MoSe_2_^38^. This was done by first assembling mechanically exfoliated flakes of graphene and hBN with the dry-transfer method, and placing them on a quartz substrate. After thermal annealing of the two-flake stack, platinum contacts were defined with e-beam lithography and deposited on top of the hBN flake through thermal evaporation (1 nm Cr + 19 nm Pt). The partially complete device was then thermally annealed again, before assembling mechanically exfoliated MoSe_2_, hBN and graphene flakes and placing them on top of the contacts. Finally, extended electrical contacts to the Pt contacts and the graphite gates were deposited through thermal evaporation (10 nm Cr + 90 nm Au).

### Experimental method

All measurements were conducted in a Montana Instruments cryostat, using a custom-built 4 f confocal setup with a Zeiss (100x, NA = 0.75, WD = 4 mm) objective. Reflection spectra were measured using a halogen source and a spectrometer, and all spectra were normalized to that collected from a gold contact. Electrostatic gating was performed with Keithley 2400 multimeters for DC measurements and with an arbitrary waveform generator (Tektronix AWG710) for AC measurements. We used a Ti:Sapphire laser (M Squared) with a power of 5 μW at the sample for Fourier imaging, and imaged the reflected beam with a CMOS camera in the Fourier plane. At high frequencies, an avalanche photodetector (APD) was used to collect photons from two different parts of the Fourier plane. The time dependence was measured using a Time Correlated Single-Photon Counting system (PicoHarp 300).

## Supplementary information

Supplementary information

Supplementary Video S1 (.mov format)

Supplementary Video S1 (.mp4 format)

## Data Availability

All data needed to evaluate the findings in the paper are present in the paper and the supplementary materials.
