# Supplementary material for: Beam steering at the nanosecond time scale with an atomically thin reflector
Source: Nat Commun. 2022 Jun 14;13:3431. doi: 10.1038/s41467-022-29976-0 (PMC9198240; doi:10.1038/s41467-022-29976-0)
Supplement: Supplementary file 1 — Supplementary information [file 41467_2022_29976_MOESM1_ESM.docx]

Supplementary Materials for

**Beam steering at the nanosecond time scale**

**with an atomically thin reflector**

Trond I. Andersen^1^, Ryan J. Gelly^1^, Giovanni Scuri^1^, Bo L. Dwyer^1^, Dominik S. Wild^1,2^, Rivka Bekenstein^1,3^, Andrey Sushko^1^, Jiho Sung^1,4^, You Zhou^1,4,5^, Alexander A. Zibrov^1^, Xiaoling Liu^1^, Andrew Y. Joe^1^, Kenji Watanabe^6^, Takashi Taniguchi^7^, Susanne F. Yelin^1^, Philip Kim^1,8^, Hongkun Park^1,4,†^, Mikhail D. Lukin^1,†^

^1^Department of Physics, Harvard University, Cambridge, MA 02138, USA

^2^Max Planck Institute of Quantum Optics, Hans-Kopfermann-Straße 1, D-85748 Garching, Germany

^3^ITAMP, Harvard-Smithsonian Center for Astrophysics, Cambridge, MA 02138, USA

^4^Department of Chemistry and Chemical Biology, Harvard University, Cambridge, MA 02138, USA

^5^Department of Materials Science and Engineering, University of Maryland, College Park, MD, USA

^6^Research Center for Functional Materials, National Institute for Materials Science, 1-1 Namiki, Tsukuba 305-0044, Japan

^7^International Center for Materials Nanoarchitectonics, National Institute for Materials Science, 1-1 Namiki, Tsukuba 305-0044, Japan

^8^John A. Paulson School of Engineering and Applied Sciences, Harvard University, Cambridge, MA 02138, USA

^†^To whom correspondence should be addressed: [lukin@physics.harvard.edu](mailto:lukin@physics.harvard.edu) and [hongkun_park@harvard.edu](mailto:hongkun_park@harvard.edu)

**Contents:**

Supplementary Notes I-VIII

Supplementary Figs. S1 to S13

**Other Supplementary Materials for this manuscript include the following:**

Supplementary Video S1

1. **Phase extraction through spectrum fitting**

The interference between the reflection from the excitons and from other interfaces within our system causes an asymmetric line shape. The resultant reflection arises from the infinite sum of optical paths created by different combinations of reflection and transmission at the various interfaces. By assuming that the background reflection in the absence of excitonic response is constant ($\left| r_{0} \right|e^{i\phi_{0}})$ in the small spectral range considered in our fits, the reflection can be written on the form^1^:

$$\begin{aligned} r\left( \omega\right)=\frac{\gamma_{0}\left( \frac{2\omega_{0}G}{c} \right)^{2}}{\omega-\omega_{0}+\frac{i\Gamma}{2}}+r_{0}=\frac{A+iB}{\omega-\omega_{0}+\frac{i\Gamma}{2}}+r_{0}, \#\left( SEQ eq \backslash* MERGEFORMAT 1 \right) \end{aligned}$$

where $\gamma_{0}$ is the free-space radiative rate of the excitons, $\Gamma$ is the total linewidth, and $\omega_{0}$ is the exciton resonance frequency. $G$ is the Green’s function that propagates fields from the TMD to the far-field, and incorporates the effects of the background reflectors. We therefore stress that the first term is not equivalent to the reflection of a freestanding TMD. Because *G* is generally complex, we replace the numerator of the first term with a complex number $A+iB$*,* where $A$ and $B$ are real.

Introducing $\delta=\omega-\omega_{0},$we find expressions for the amplitude and phase of the reflection:

$$\begin{aligned} R\left( \omega\right)=\left| r\left( \omega\right) \right|^{2}=\left| r_{0} \right|^{2}&+\frac{A^{2}+B^{2}+B\Gamma\left| r_{0} \right|+2A\left| r_{0} \right|\delta}{\delta^{2}+\frac{\Gamma^{2}}{4}}\#\left( SEQ eq \backslash* MERGEFORMAT 2 \right) \end{aligned}$$

$$\begin{aligned} \phi\left( \omega\right)-\phi_{0}=\tan^{-1} \left( \frac{B\delta-\frac{A\Gamma}{2}}{A\delta+\frac{B\Gamma}{2}+|r_{0}|(\delta^{2}+\frac{\Gamma^{2}}{4})} \right)\#\left( SEQ eq \backslash* MERGEFORMAT 3 \right) \end{aligned}$$

By fitting the obtained reflection spectra, we extract the parameters $|r_{0}|$, $A$, $B$, $\delta$ and $\Gamma$, and can thus compute the phase. Since we are only concerned with relative phases in our work, we set $\phi_{0}=0$.

1. **Effects of background reflections**

Interestingly, while the background reflections typically reduce the phase range to a value below $\pi$ (range of the pure exciton resonance), they can actually also increase the phase range to $2\pi$ if their phase and amplitude are chosen correctly. Hence, the phase range is not inherently limited in our approach. To understand how this is possible, we write the total reflection as:

$r\left( \omega\right)=\frac{C}{2(\omega-\omega_{0})/\Gamma+i}+r_{0}$,

where $\Gamma$ is the total exciton linewidth, $\omega_{0}$ is the exciton resonance frequency and $r_{0}=\left| r_{0} \right|e^{i\phi}$ is the background reflection that would be observed in the absence of excitons. We note that $C$ is also complex. Crucially, while the phase of the first term only varies by $\pi$ across the resonance, the phase of the full sum can vary by $2\pi$. This can be intuitively understood by considering the development of $r\left( \omega\right)$ in the complex plane as shown in Fig. S1c: as $\omega$ is moved past the resonance, the reflection traces out a circle with radius $|C|/2$ centered at $r_{0}-iC/2$. (In reality, the slight frequency dependence of the background reflection causes a small deformation of this circular path). Hence, in order to achieve the desired $2\pi$ phase range, this circle needs to encompass the origin, which is the case so long as $\left| r_{0}-\frac{iC}{2} \right|<|C|/2$.

To support this theoretical picture, we show reflection spectra from additional, ungated devices demonstrating the full $2\pi$ phase range across the exciton resonance (Fig. S1), through more optimized background reflections and stronger TMD reflection in those devices. As can be seen from Fig. S1c, the reflection traces encompass the origin in the complex plane, indicating the full $2\pi$ phase range (also evident in Fig. S1b). However, further optimization is required to make the circle more centered at the origin to prevent large variations in reflection amplitude, as can be seen in Fig. S1a.

In order to understand how the effects of background reflections depend on hBN thickness and substrate, we conducted a detailed quantitative analysis of the reflections within our device structure, using the transfer matrix method. This theoretical model is described in further detail in Ref. ^1^, and reproduces our experimental observations very well, as can be seen in Fig. S1a. We note that our model accounts for not only the effect of hBN thickness on $r_{0}$, but also on $C$, which depends on the Green’s function that propagates fields through the heterostructure. Fig. S2e-h shows the calculated phase range for a wide range of hBN thicknesses and several substrate types (Fig. S2a-d). Crucially, our analysis demonstrates that a phase range of $2\pi$can be achieved in a substantial part of the parameter space. We also show the maximum reflection amplitude in Fig. S2i-l, and the relative amplitude variations in Fig. S2m-p. Achieving optimal operation also requires simultaneously maximizing the reflection amplitude and minimizing its variation across the resonance. Fig. S2 shows that the use of a gold reflector under the device (Fig. S2c,d) can be a promising way to achieve desirable values for all the three criteria simultaneously.

Interestingly, as shown in the rightmost inset of Fig. S1c, the background reflections can in principle be optimized such that $r_{0}=\frac{iC}{2}$, causing the reflection trace to form a circle that is centered at the origin in the complex plane. Notably, in this case, the reflection amplitude stays constant across the whole exciton resonance. Thus, the exciton-based approach shown in our work could in principle allow for making pixels with both the full phase range of $2\pi$ and constant reflection. For instance, Fig. S3 shows the numerically calculated reflection of a device placed on a gold covered substrate with top and bottom hBN-thicknesses of 28 and 40 nm, respectively. (Note that a thin hBN layer on top of the gold as shown in Fig. S2c would prevent shorting bottom graphene gates.) To here evaluate the optimal case, we have used the exciton properties of device C, which has a stronger reflection than devices A and B. (Note that this is not necessary to achieve constant reflection amplitude but increases the constant reflection amplitude). Notably, we find a full phase range of $2\pi$, reflection in excess of 84%, and near constant (<1% variation) reflection amplitude. We note that the optimal hBN thicknesses and choice of substrate depend on the exact radiative and non-radiative rates of the excitons, which vary from device to device. We therefore emphasize that while our quantitative analysis provides a deeper understanding of the effects of background reflections and demonstrates that our system is not inherently limited with regards to phase range and reflection amplitude, further studies are required to experimentally achieve complete optimization. A promising avenue for this task is utilizing “feedback fabrication” schemes where additional hBN layers are added after characterizing the TMD. Another appealing path involves the use of a substrate with globally tunable refraction index. It should be stressed that the refractive index of such a substrate would only need to be fine-tuned once and only on a global level, and would therefore not require the fast, local tunability demonstrated with a TMD in our work.

1. **Gate edge localization**

In order to position the laser beam at the gate edge, we first locate the edge by sweeping the galvanometric mirrors while measuring the reflection using a broadband halogen lamp. To further optimize the alignment of the laser spot with the gate edge, we measure beam steering in a few locations near the gate edge (example shown in Fig. S4). The relative contributions from the two sides vary as the spot is moved across the edge, and we balance the contributions by finding the location with the largest and most symmetric deflection range. Far away from the gate edge, only small deflections (in arbitrary directions) are observed (Fig. S5a,b), likely due to local inhomogeneity, causing small phase gradients that change slightly with doping. The non-zero width of the deflection path at the gate edge is likely caused by such inhomogeneity. Independent control of different parts of the wavefront, essential to amplitude stabilization (Fig. 2f in main text), two-dimensional steering and further upscaling possibilities, is of course only possible at the split-gate edge.

1. **Theoretical modeling of beam deflection**

We here present a theoretical model of the beam deflection, the predictions of which are in very good agreement with the experimental observations presented in the main text. Defining the gate edge to lie along $x=0$, we model the reflection profile of our system as:

$$\begin{aligned} r\left( \omega,\boldsymbol{r} \right)\boldsymbol{=}\left\{ \begin{aligned} r_{\mathrm{tot}\boldsymbol{,}L}\left( \omega\right)\delta\left( z \right), x<0 \\ r_{\mathrm{tot}\boldsymbol{,}R}\left( \omega\right)\delta\left( z \right), x>0, \end{aligned} \right.\#\left( SEQ eq \backslash* MERGEFORMAT 4 \right) \end{aligned}$$

where $r_{\mathrm{tot}\boldsymbol{,}L(R)}\boldsymbol{(}\omega)$ is the (gate-dependent) combined spectrum of the exciton and background reflections on the left (right) side of the gate edge. The combined spectrum has a smaller available phase range than that of the exciton resonance itself (0-180$^{\circ}$) due to the interference with the background reflections. While the background reflections come from multiple interfaces both above and below the TMD, the combined system can be modeled as an equivalent reflector in the plane of the TMD ($z=0)$by including the additional phases due to the *z*-displacements in $r(\omega)$.

The incoming field is given by the two-dimensional Gaussian distribution $E\left( \rho\right)=E_{0}exp(-\rho^{2}/4\sigma^{2})$, where $\rho$ is the radial coordinate in the plane of the TMD and $\sigma$ is the standard deviation of the incoming intensity distribution, $I_{\mathrm{in}}\left( \rho\right)=c\varepsilon\left| E\left( \rho\right) \right|^{2}$. For a diffraction limited spot, one finds $\sigma=0.42\lambda/(2\mathrm{NA})$, where *NA* is the numerical aperture of the objective^2^. The reflected intensity at a position $\boldsymbol{r}$ is then given by:

$$\begin{aligned} I\left( \boldsymbol{r,}\omega\right)={c\varepsilon\left| \int r\left( \omega,\boldsymbol{r}^{\boldsymbol{'}} \right)E\left( \boldsymbol{r}^{\boldsymbol{'}} \right)\frac{\exp\left( ik\left| \boldsymbol{r-}\boldsymbol{r}^{\boldsymbol{'}} \right| \right)}{\lambda\left| \boldsymbol{r-}\boldsymbol{r}^{\boldsymbol{'}} \right|}d\boldsymbol{r}^{'} \right|}^{2},\#\left( SEQ eq \backslash* MERGEFORMAT 5 \right) \end{aligned}$$

Computing the integral for the far-field ($r\to\infty$) at a polar angle $\theta$, and azimuthal angle $u$ ($u$=0 is perpendicular to the gate edge), one finds:

$I\left( \theta\boldsymbol{,}u,\omega\right)\propto\exp\left( {-2F}^{2}\left( \theta\right) \right)\left| r_{tot,R}\left( \omega\right)\left[ 1-i\mathrm{erfi}\left( F\left( \theta\right)\cdot\cos u \right) \right]+r_{tot,L}\left( \omega\right)\cdot\left[ 1+i\mathrm{erfi}\left( F\left( \theta\right)\cdot\cos u \right) \right] \right|^{2},$ $( SEQ eq \backslash* MERGEFORMAT 6)$

where $F\left( \theta\right)=k\sigma\cdot\sin\theta$, and $erfi(x)$is the imaginary error function. Thus, we find that the reflections from the two sides interfere constructively for $\theta,u$ that satisfy:

$$\begin{aligned} \theta=\sin^{-1} \left( \frac{\mathrm{erfi}^{-1} (\tan\Delta\phi/2)}{k\sigma\cos u} \right),\#\left( SEQ eq \backslash* MERGEFORMAT 7 \right) \end{aligned}$$

where $\Delta\phi$ is the phase difference between the reflections from the two sides. In the small phase difference limit, this simplifies to $\theta=\Delta\phi\cdot\sqrt{\pi}/(4k\sigma\cos u)$, equivalent to two localized sources at $x=\pm2\sigma/\sqrt{\pi}$. Due to the factor $\exp\left( {-2F}^{2}(\theta) \right)$, the intensity maximum appears at a somewhat different angle. In cases where the amplitudes of the two reflections can be approximated to be the same, the integral simplifies to (in the small $\Delta\phi$ limit):

$$\begin{aligned} I\left( \theta\boldsymbol{,}u,\omega\right)\propto{4e}^{-{2F}^{2}(\theta)}\left| r_{\mathrm{tot}}\left( \omega\right) \right|^{2}\left( 1+\mathrm{erfi} (F(\theta)\cdot\cos u )\cdot\Delta\phi\right),\#\left( SEQ eq \backslash* MERGEFORMAT 8 \right) \end{aligned}$$

Since the maximum intensity appears at small $\theta$ in the small $\Delta\phi$ limit, and $\mathrm{erfi}\left( x \right)\sim2x/\sqrt{\pi}$ for small $x$, we find:

$$\begin{aligned} \left( u,\theta\right)_{\max}=\left( 0,\frac{\Delta\phi}{2\sqrt{\pi}k\sigma} \right),\#\left( SEQ eq \backslash* MERGEFORMAT 9 \right) \end{aligned}$$

Finally, the center-of-mass deflection perpendicular to the gate edge is found by using the Taylor expansion of $erfi(x)$, since the integral requires evaluating $erfi(x)$ up to $x=k\sigma\sin\left( \theta_{c} \right)$ where $\theta_{c}$ is the collection angle:

$$\begin{aligned} \boxed{\bar{\theta}_{\perp}=\bar{\theta}_{x}=\frac{\int I\theta\cos\left( u \right)d\Omega}{\int Id\Omega}=\frac{\Delta\phi}{\sqrt{2\pi}k\sigma}=\frac{\Delta\phi\cdot\mathrm{NA}}{0.42\cdot\sqrt{2\pi^{3}}}}\#\left( SEQ eq \backslash* MERGEFORMAT 10 \right) \end{aligned}$$

In solving the integrals, we have made the approximation $\sin\theta\sim\theta$, which is an acceptable approximation when $k\sigma>1$, since the exponential factor then suppresses terms at large $\theta$. For a diffraction-limited spot, one finds $k\sigma\sin\theta_{c}= k\sigma\cdot\mathrm{NA}=1.3,$or $e^{-2\left( k\sigma\sin\theta_{c} \right)^{2}}=0.03$.

We compare eqn. (10) with the exact (numerically solved) deflection in Fig. S6 for NA=0.75 ($k\sigma\sim1.8)$, and find that they are very similar. Moreover, the theoretically predicted deflection range of $\sim$10$^{\circ}$ ($\pm5^{\circ}$) for a phase difference range of 42$^{\circ}$ is in excellent agreement with our experimental observations.

1. **High-frequency transmission characterization**

The high-frequency measurements presented in Fig. 4d in the main text show clear beam steering down to switching times of 1.6 ns ($\omega=2\pi\cdot316$MHz). However, the amplitude is found to decrease at the highest frequencies, which could be due to either the *RC*-time of the device itself or the external cabling leading up to the device. In order to determine the impact of the latter, we use a Vector Network Analyzer (VNA) to characterize the high-frequency performance of parts of the external cabling that was used in the measurement (Fig. S7). While we cannot recreate the full circuit in the absence of the device without introducing additional components, this measurement places an upper bound on the transmission of the full cabling. We find a substantial reduction in the transmission at the higher frequencies used in our work, with S12 parameters of 50% and 34% at $\omega=2\pi\cdot178$MHz and $\omega=2\pi\cdot316$MHz, respectively. This gives a lower bound on the decay factor due to the device itself of 75% and 61% at the two frequencies, respectively.

1. **Temperature dependence**

Fig. S8a shows reflection spectra obtained in the right side of the device in the intrinsic regime at five different temperatures ranging from 6 K to 300 K. Consistent with previous studies^1^, we find that the exciton resonance broadens, decreases in amplitude, and red-shifts with increasing temperature. While the phase of the exciton resonance still changes from 0$^{\circ}$ to 180$^{\circ}$ across the resonance, the decrease in amplitude causes a reduction in the available phase range of the combined background and exciton signal (Fig. S8b). However, we find that the reflection amplitude and phase range remain almost the same at liquid nitrogen temperatures (80 K) as at 6 K, and the exciton resonance is still clearly visible at higher temperatures.

Since the resonance wavelength changes with temperature, we conduct gate-dependent beam steering measurements for a range of laser wavelengths at each temperature to ensure proper comparison across temperatures (Fig. S8c), and present scatter plots from the optimal wavelengths in Fig. S8d. The deflection ranges shown in Fig. S8c are based on the set of gate voltage combinations used in the main text. Consistent with the reduction in phase range, the beam deflection range decreases with temperature (Fig. S8c,d). Nevertheless, the deflection range remains almost 8$^{\circ}$ at 80 K and 2$^{\circ}$ at 150 K. Some deflection of the full reflection can also be observed at 230 K (-43 $℃$) and even at room temperature (Fig. S8d). As discussed in Supplementary Note II, the operation at these higher temperatures could be improved by optimizing the background reflections, thus allowing for a larger phase range of the combined reflection.

1. **Upscaling to more pixels**

We here discuss two realistic avenues for upscaling: first, through standard etching techniques, the gates can be divided into very many pixels with sub-100 nm dimensions and be employed in the same way as demonstrated in our current work. One appealing design enabled by our use of gates in two different planes is to etch the top and bottom gates into long, perpendicular strips (Fig. S9). This way, the gate strips form a two-dimensional grid of pixels, where the phase of pixel (*i,j*) is set by the voltages applied to top gate strip *i* and bottom gate strip *j*, through the same double capacitor mechanism as in our current work. While such a design enables independent control of $2n$ degrees of freedom for an $n\times n$-grid, applications that would require more independent channels could be achieved through further reductions in the pitch of nanoelectrode arrays. By placing our device structure on top of such arrays, full independent control of all pixels could be achieved. Since the mechanism behind such control would still be electrostatic doping, the switching rates are expected to be similar to the ones demonstrated in our current work. Through scaling up the device to more pixels, more complex beam shaping can be achieved, and a larger incident beam spot (smaller beam divergence angle) can be used.

1. **Polarization dependence**

In order to test how the performance of our beam steering device depends on the polarization of the incoming light, we measure the amplitude and deflection of the reflection using four different (linear) polarization angles (Fig. S10). To remove any effects of polarization-dependent optical components in the beam path (e.g. beam splitters), we normalize the amplitude of the reflection to that without excitonic effects, obtained by heavily doping the device. Since the latter is not affected by gradients in exciton properties, we assume it to be an isotropic reference. Fig. S10a-d shows the gate dependence of the integrated reflection, indicating no systematic variations with polarization angle. Plotting the gate dependence of the deflection angle (Fig. S10e-h) and a scatter plot of all deflections (Fig. S10i-l), we also observe no systematic polarization dependence of the deflection range or the deflection direction. The small (non-systematic) variations observed in Fig. S10 are expected to be due to small changes in spot location as the polarization very weakly affects the beam path. The robustness to polarization variations stands in contrast to many other types of beam steering devices and is a very useful feature for many applications.


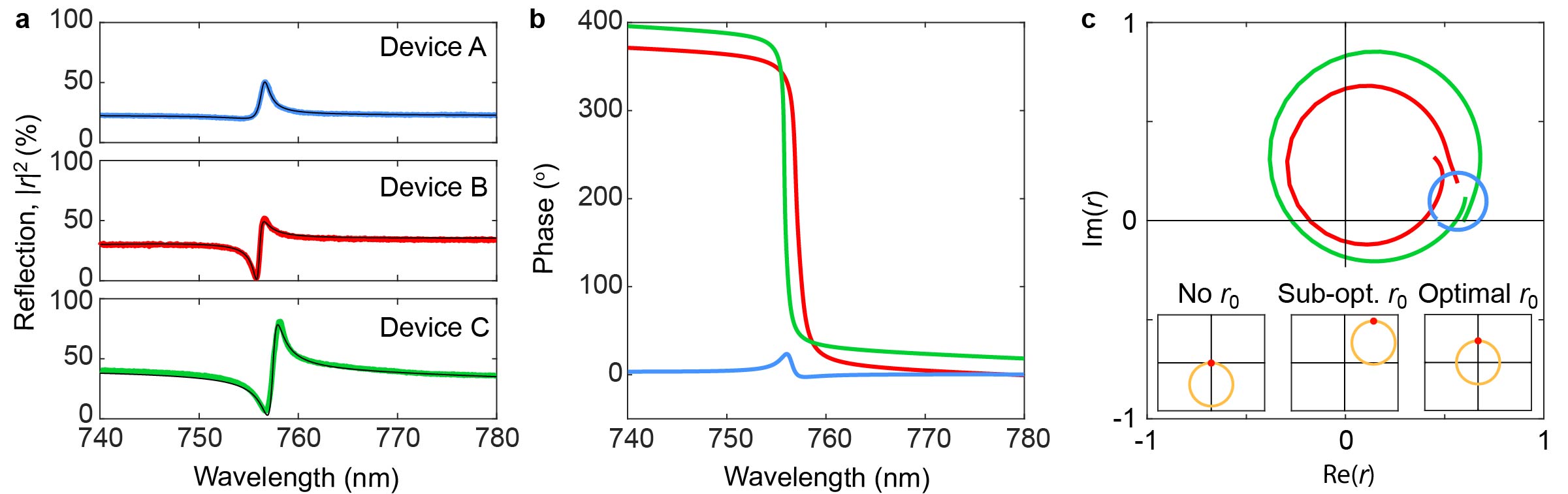


1. **:** **Demonstration that** $\boldsymbol{2}\boldsymbol{\pi}$ **phase range is possible in the exciton-based approach. a,** Reflection spectra (colored curves) with fits based on the transfer matrix method (black) for devices A, B and C (top to bottom). Top/bottom hBN thicknesses and substrates for the three devices are, A: 8 nm/60 nm, quartz; B: 70 nm/100 nm, Si/SiO_2_; C: 55 nm/86 nm, Si/SiO_2_. **b**, Phase extracted from fits in **a**, demonstrating phase range of $2\pi$ across exciton resonance in devices B and C. **c**, Complex plane representation of reflection parametrized by wavelength, demonstrating that the reflection traces a circle around the origin in devices B and C. Insets: Illustrations of complex $r$ in cases with zero, sub-optimal and optimal background reflection, giving phase ranges of $\pi,$ $<\pi$ and $2\pi$, respectively. Red dot indicates $r_{0}$. Without loss of generality, the parameter *C* is set to be real.


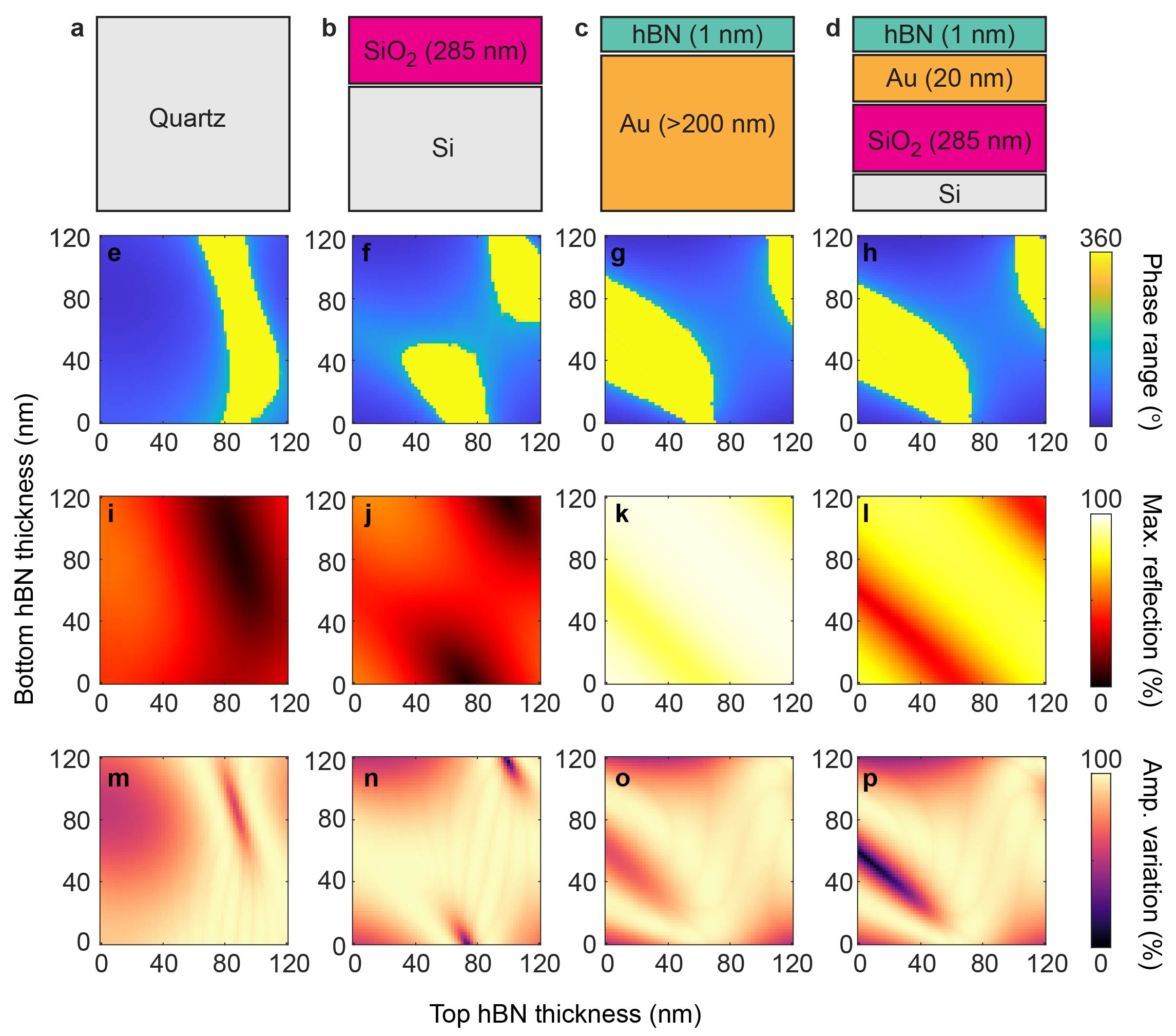


1. **:** **Effect of hBN thickness and substrate on phase range, reflection and amplitude stability. a-d,** Schematics of different substrate options. A thin (1 nm) hBN layer is included in **c** and **d** since the gold would otherwise short the bottom graphene gates. **e-h**, Phase range as a function of hBN thicknesses for the four substrate options, demonstrating that a phase range of $2\pi$ is achievable in a substantial part of the parameter space. **i-l**, Maximum reflection amplitude. **m-p,** Relative amplitude variation. Simultaneous optimization of all three characteristics could be achieved using substrates that include a gold layer (**c**,**d**).


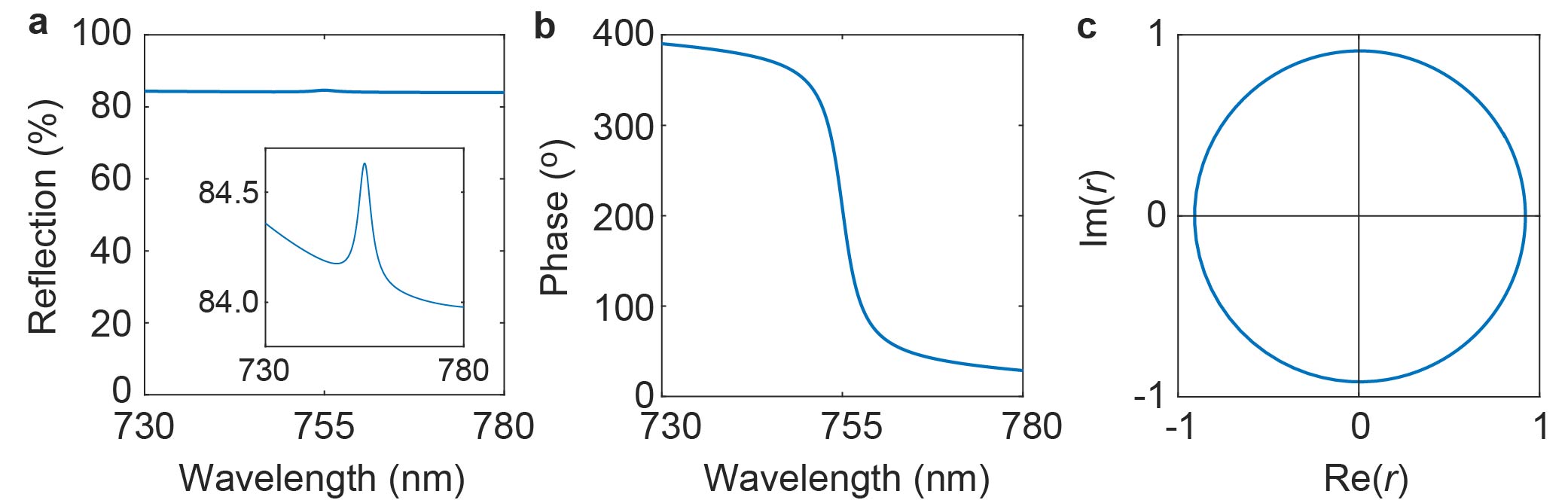


1. **:** **Performance with optimized device design. a,** Numerically calculated reflection spectrum of device on gold-covered substrate with top and bottom hBN thicknesses of 28 and 40 nm, respectively. A large ($\sim84\%)$ reflection amplitude is achieved while keeping the variation smaller than 1% in this optimized design. Inset: Zoomed in version of the main plot. **b**, Phase of reflection, showing $2\pi$ range across the resonance. **c,** Complex plane representation of reflection parametrized by the wavelength, demonstrating that the circle is very close to being centered at the origin.

**
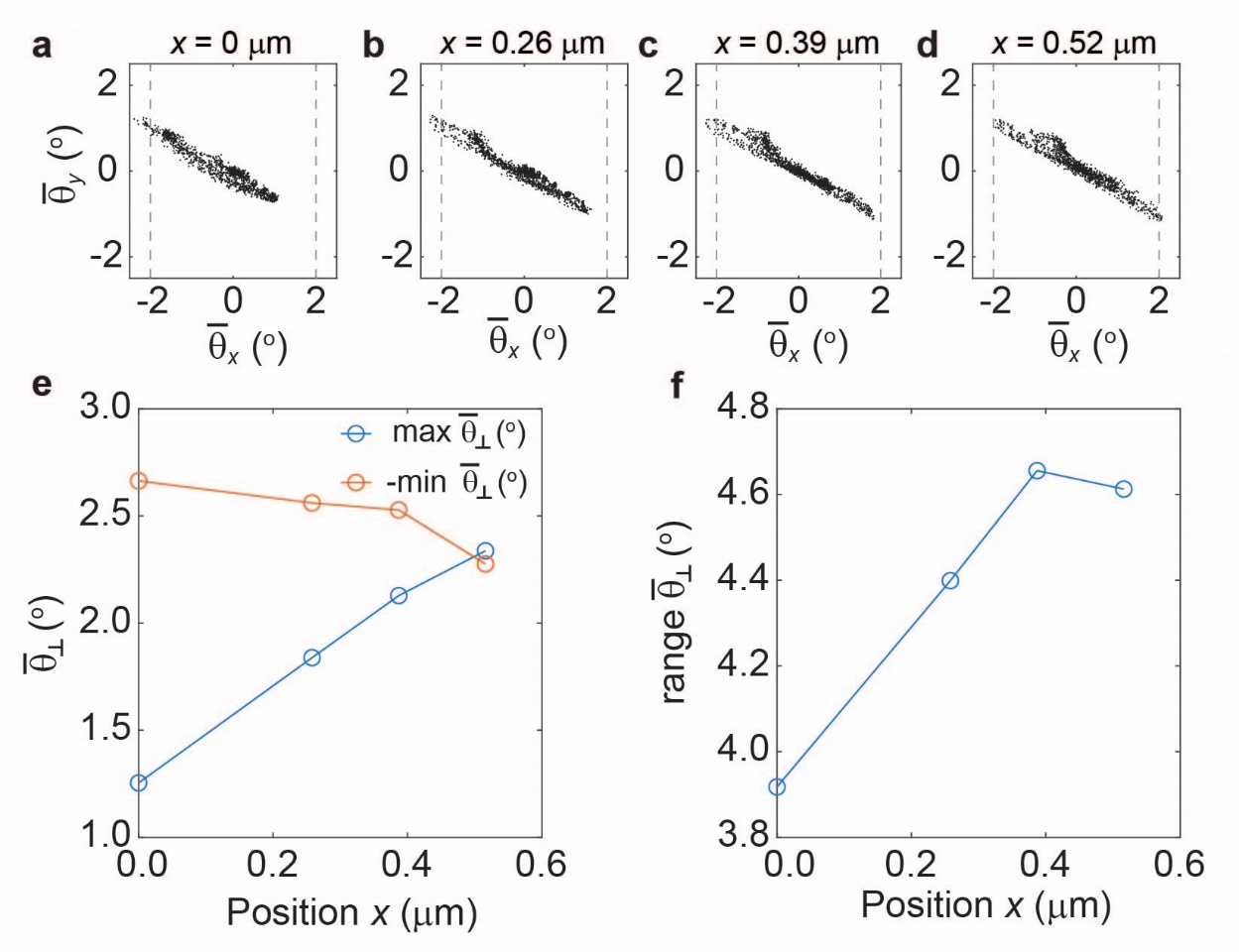
**

1. **: Example of gate edge localization.** **a**-**d**, Scatter plot of center-of-mass deflection ($\bar{\theta}_{x}$, $\bar{\theta}_{y}$) for the same set of gate voltages as in the main text in four different positions along a line crossing the gate edge ($\lambda_{0}$=758.4 nm, *T*=80 K). (*x*=0 is arbitrarily defined). **e**,**f**, Position dependence of the extrema (**e**) and range (**f**) of the beam deflection perpendicular to the gate edge. The contributions from the two sides vary across the gate edge, and are balanced by making the deflection range as large and symmetric as possible (around *x*=0.4$\mu$m).


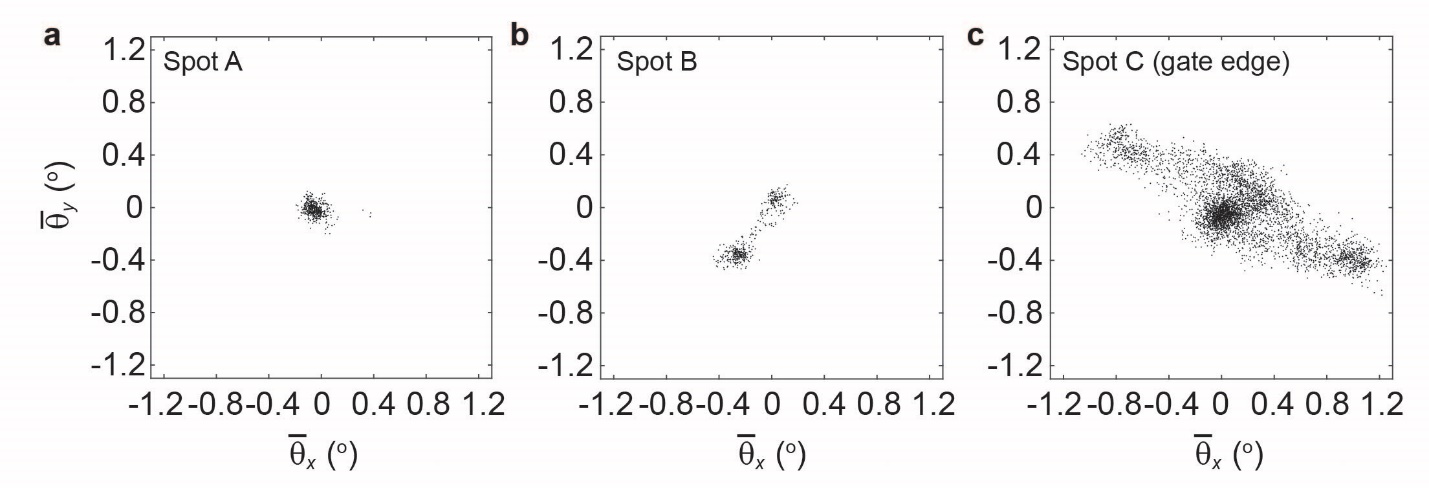


1. **: Deflection behavior away from gate edge.** **a**,**b**, Scatter plot of center-of-mass deflection ($\lambda_{0}$=754.5 nm) in two spots that are 2 $\mu$m to the left and right of the gate edge, respectively. All doping regimes are covered by sweeping the full range of the (global) top gate ($0 V<V_{\mathrm{TG}}<1.4 V$). The deflection is found to be very small far away from the gate edge, with only small deviations in arbitrary directions (example shown in **b**), likely caused by inhomogeneity. Inhomogeneity is likely the cause of the non-zero width of the deflection path at the gate edge. **c**, Scatter plot of center-of-mass deflection ($\lambda_{0}$=754.5 nm) with the beam spot centered at the gate edge, for the same set of gate voltages as in the main text. Measurements in **a**-**c** are performed with $\sim2.5$x enlarged spot-size, as in Fig. S11b.


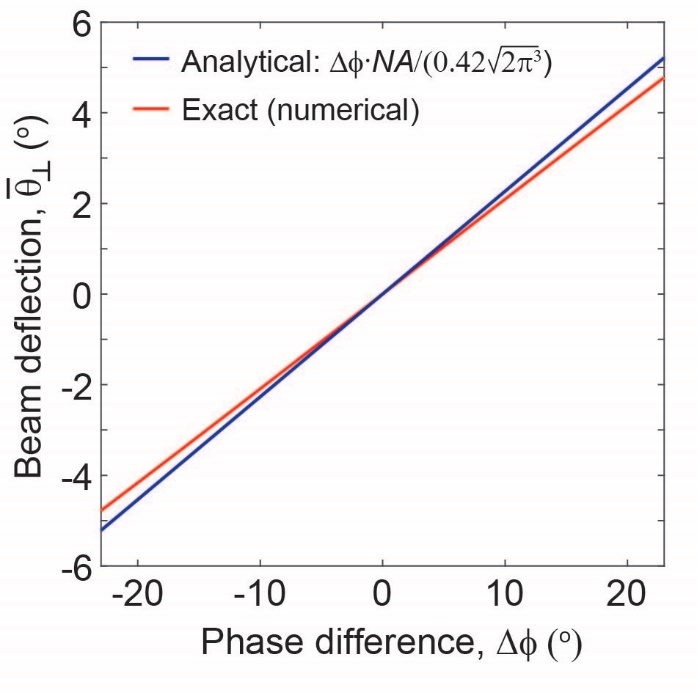


1. **: Theoretically predicted beam deflection.** Analytically (blue) and numerically (red) predicted beam deflection as a function of phase difference between the two sides of the gate edge. The predicted beam deflection range of approximately $10^{\circ}$ $(\pm5^{\circ})$ for a phase range of $42^{\circ}$ is in excellent agreement with the experimental results presented in the main text.


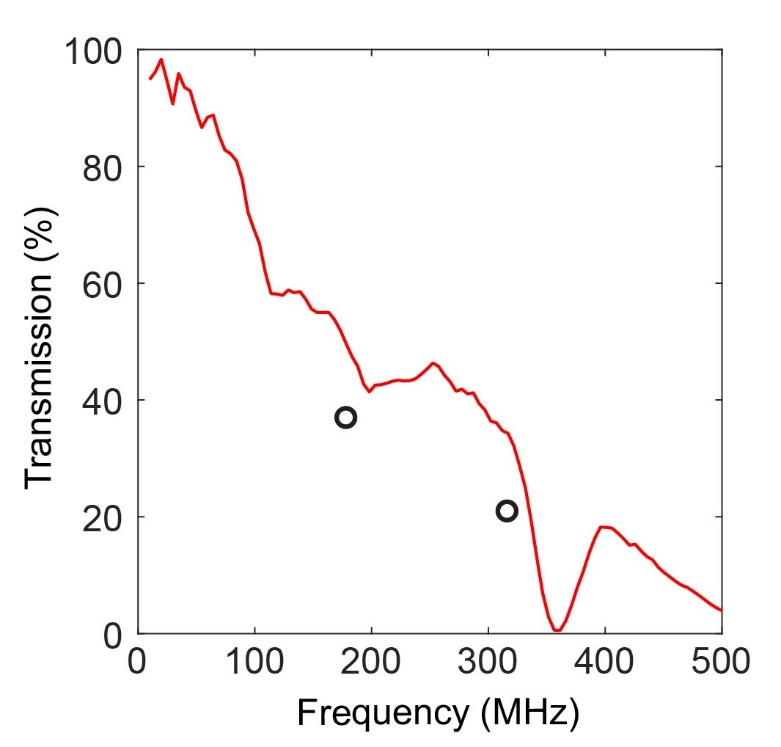


1. **: High-frequency transmission.** Red curve: VNA measurement of transmission (S12 parameter) through parts of the cabling (excluding the device) used to measure the high-frequency data in Fig. 4d. Black circles: Normalized oscillation amplitude from Fig. 4d in the main text, averaged for $\theta_{x}>0$ and $\theta_{x}<0$.


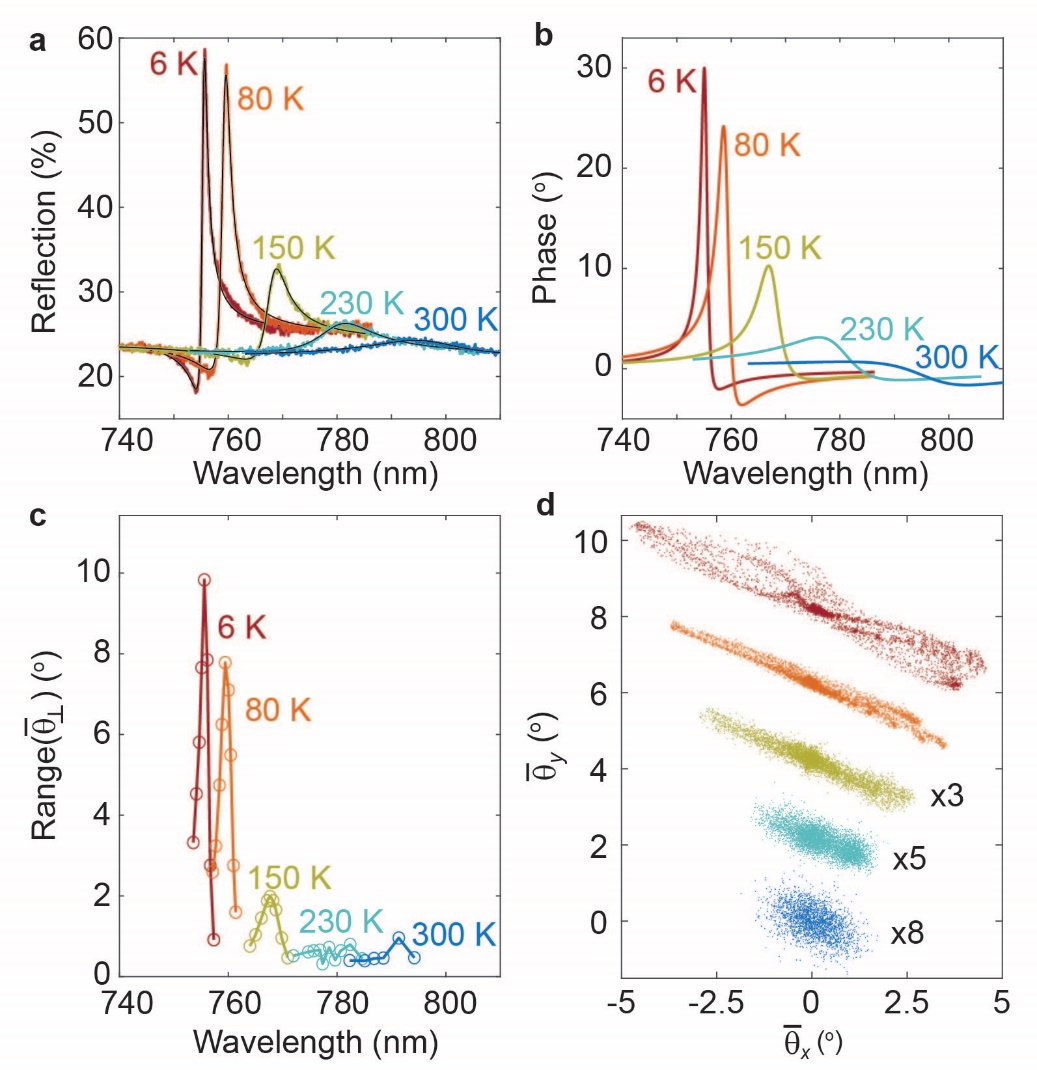


1. **:** **Temperature dependence.** **a**, Reflection spectra at temperatures 6 K (maroon), 80 K (orange), 150 K (yellow), 230 K (teal) and 300 K (blue), showing line broadening, amplitude reduction and red-shift of the exciton resonance with increasing temperature. Black: fits. **b**, Phase calculated from fits in **a**. The decrease in exciton reflection amplitude at higher temperatures reduces the available phase range of the combined exciton and background reflection. **c**, Beam deflection range perpendicular to the gate edge at the same temperatures as in **a** and **b** for a range of wavelengths near the exciton resonance. The deflection range is obtained for the same gate voltage ranges as used in the main text. The reduced phase range at higher temperatures causes a decrease in deflection range. However, the deflection range is almost unchanged from 6 K to liquid nitrogen temperature (80 K), and is still approximately 2$\boldsymbol{^{\circ}}$ at 150 K. **d**, Scatter plot of beam deflection for the optimal wavelength at each temperature, displaced in the vertical direction for enhanced visibility. Plots at 150 K, 230 K and 300 K are scaled by 3, 5 and 8, respectively. Beam deflection is still observable at 230 K, and some deflection is even observed at room temperature (300 K).


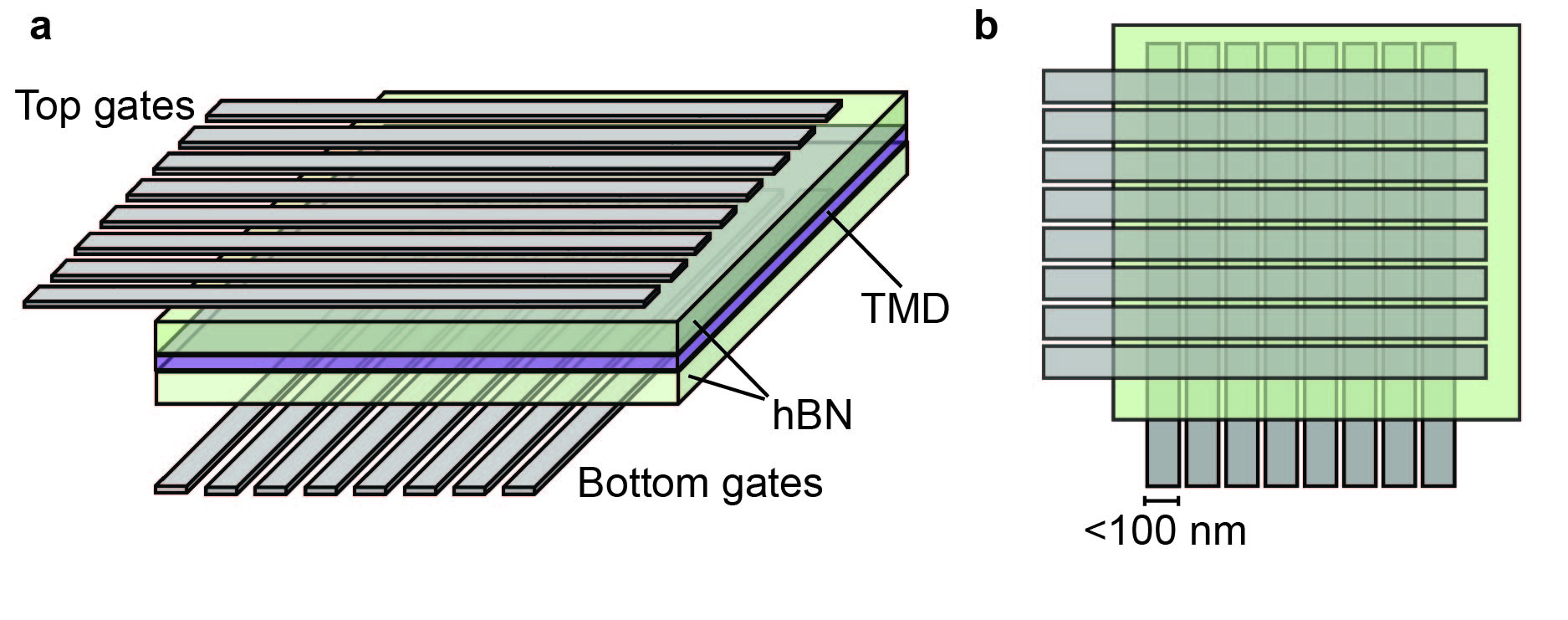


1. **:** **Upscaling potential. a,b,** Side and top-view schematics of one route for scaling up to devices with many pixels. Through standard etching techniques, the gates can be patterned into sub-100 nm wide strips, forming a 2D grid. By the same double capacitor mechanism as demonstrated in our work, the combination of voltages applied to top gate strip *i* and bottom gate strip *j* controls the phase of pixel (*i,j*).


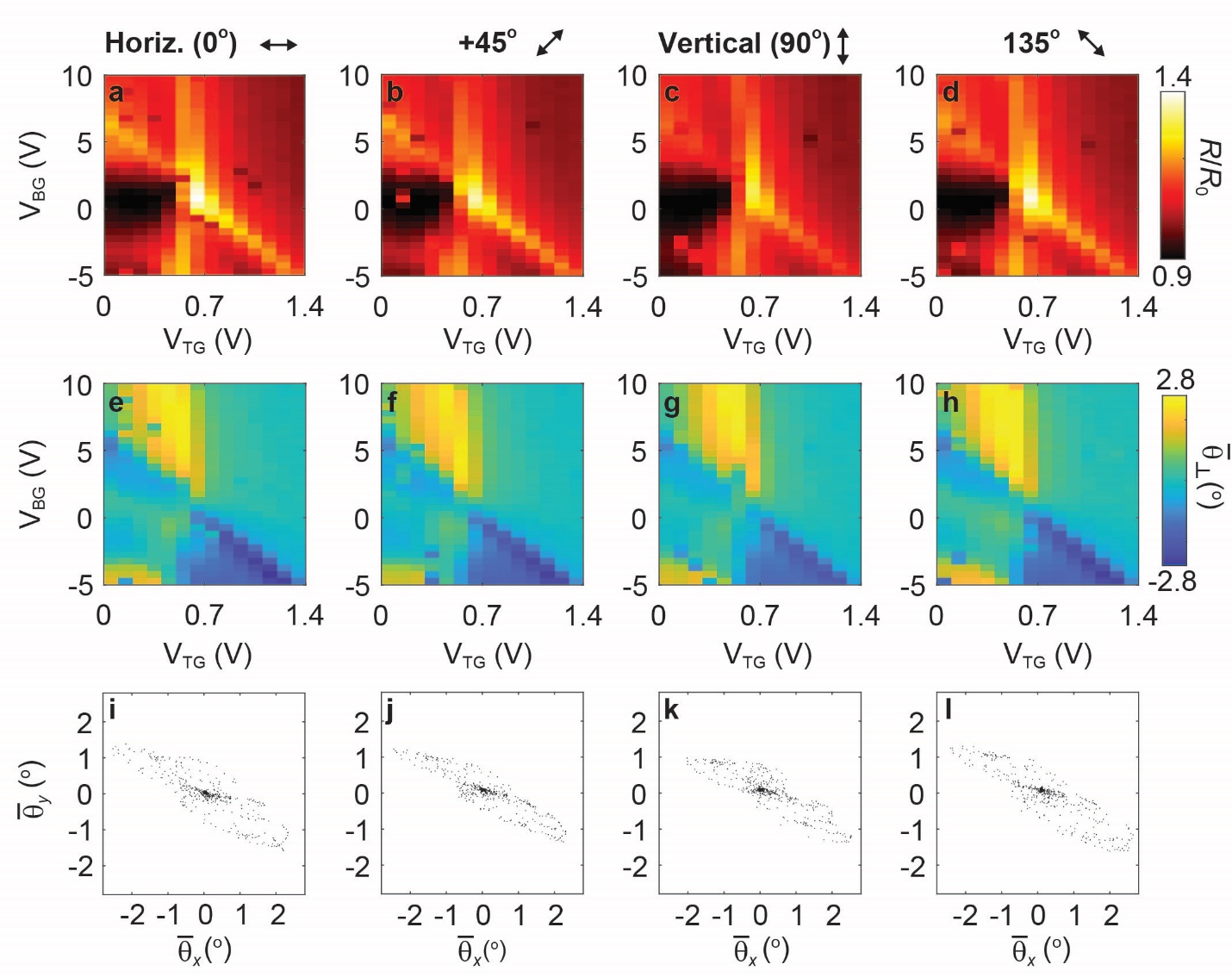


1. **:** **Polarization dependence of beam steering performance.** **a**-**d**, Gate dependence of total integrated reflection ($\lambda_{0}$=755 nm), normalized to that obtained in the highly doped regime (*V*_TG_=1.4 V, *V*_BG_=10 V), for polarization angles 0$^{\circ}$ (**a**), 45$^{\circ}$ (**b**), 90$^{\circ}$ (**c**) and 135$^{\circ}$ (**d**). **e**-**h**, Gate dependence of beam deflection perpendicular to gate edge, and (**i**-**l**) scatter plot of deflections for all gate combinations, for the same linear polarization angles as in **a**-**d**. No systematic polarization dependence of the reflection amplitude, deflection range or deflection direction is observed.


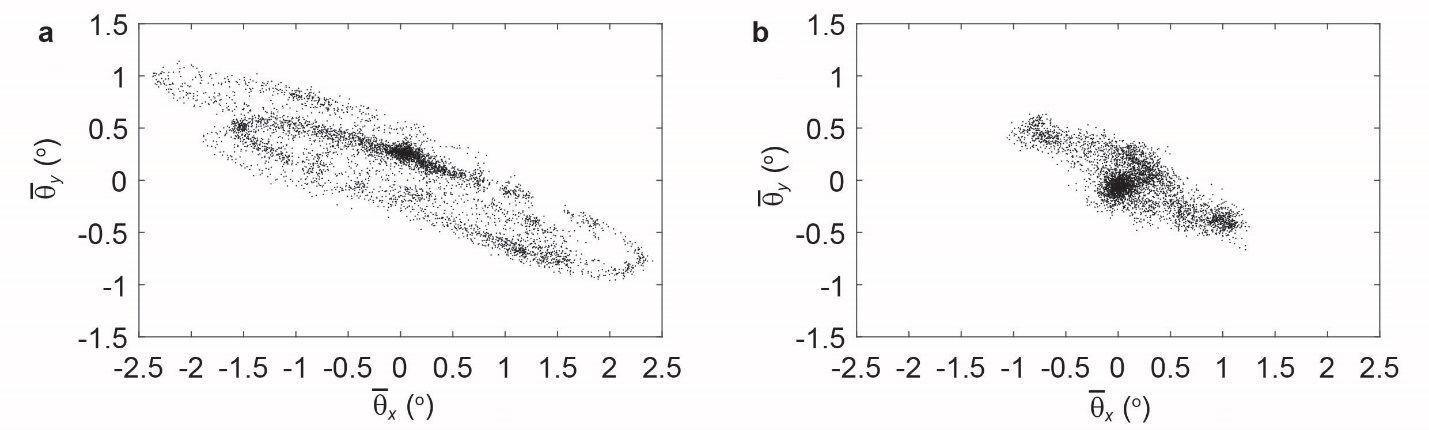


1. **: Spot size dependence.** **a**,**b**, Scatter plot of center-of-mass deflection ($\lambda_{0}$=754.5 nm) for the near-diffraction limited spot size used in the main text (**a**), and a $\sim2.5$ times larger spot size (**b**). The deflection range is found to be approximately a factor of 2 smaller for the larger beam spot (**b**), in good agreement with the predictions in Supplementary Note IV.


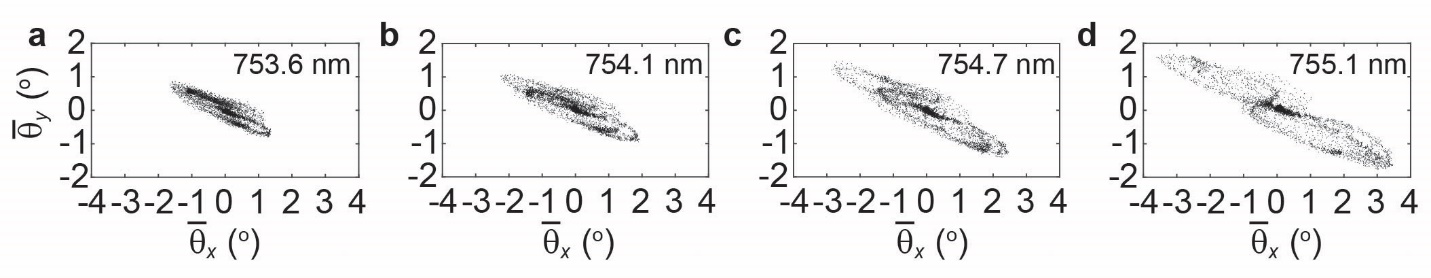


1. **:** **Beam steering at other wavelengths.** **a**-**d**, Scatter plot of center-of-mass deflection ($\bar{\theta}_{x}$, $\bar{\theta}_{y}$) for the same set of gate voltages as in the main text at $\lambda_{0}$=753.6 nm (**a**), $\lambda_{0}$=754.1 nm (**b**), $\lambda_{0}$=754.7 nm (**c**) and $\lambda_{0}$=755.1 nm (**d**). The deflection range is smaller at longer wavelengths, because the required large blue-shift is accompanied by a reduction in exciton reflection amplitude.


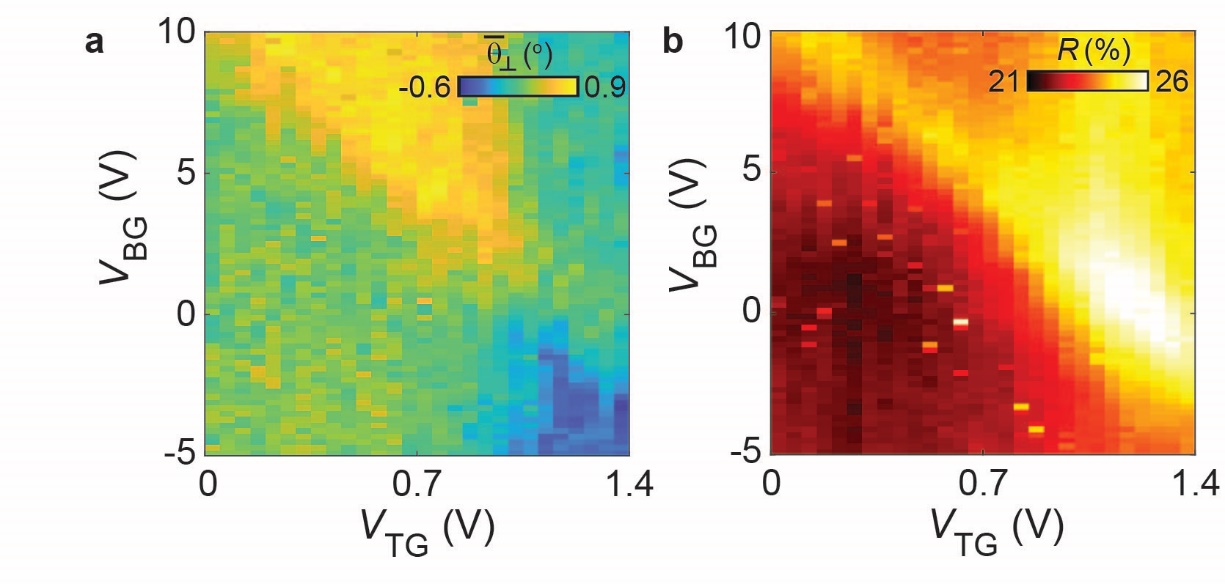


1. **:** **Beam steering at** $\boldsymbol{\lambda}_{\boldsymbol{0}}$**=752 nm.** **a**,**b**, Gate voltage dependence of deflection perpendicular to gate edge (**a**) and integrated reflection (**b**). At this short wavelength, larger voltages are required to blue-shift the exciton resonance through $\lambda_{0}$, compared to wavelengths closer to the intrinsic exciton resonance.

**References:**

1 Scuri, G. *et al.* Large Excitonic Reflectivity of Monolayer MoSe2 Encapsulated in Hexagonal Boron Nitride. *Physical Review Letters* **120**, 037402 (2018).

2 Zhang, B., Zerubia, J. & Olivo-Marin, J.-C. Gaussian approximations of fluorescence microscope point-spread function models. *Applied Optics* **46**, 1819-1829 (2007).
